# Management of Post-craniotomy Pain in Elective Cases: A Randomized Controlled Trial

**DOI:** 10.7759/cureus.46189

**Published:** 2023-09-29

**Authors:** Kumar Abhinav, Dikpal Jadhav, Uday B Andar, Vikram Karmarkar, Rama Agrawal, Ankita Agrawal

**Affiliations:** 1 Department of Neurosurgery, Lilavati Hospital and Research Centre, Mumbai, IND; 2 Department of Neurosurgery, King Edward Memorial (KEM) Hospital, Mumbai, IND; 3 Department of Neurosurgery, Bai Jerbai Wadia Hospital for Children, Mumbai, IND; 4 Department of Neurosurgery, Bombay Hospital and Medical Research Centre, Mumbai, IND; 5 Department of Physiology, Patna Medical College and Hospital, Patna, IND; 6 Department of Conservative and Endodontics, Buddha Institute of Dental Sciences & Hospital, Patna, IND

**Keywords:** post-operative pain, neurosurgery, nrs scales, eras, craniotomy

## Abstract

Background:* *Craniotomy is associated with significant postoperative discomfort. Standardized pain management and enhanced recovery after surgery (ERAS) protocol could improve patient-reported outcomes and lower medical expenses.

Aim: The aim of this study is to prospectively assess the effectiveness of an ERAS protocol for neurosurgery in the treatment of postoperative pain following elective craniotomies.

Methods and materials: A total of 128 patients were assigned to the ERAS group and received care in accordance with the neurosurgical ERAS regulations, while 130 other participants were assigned to the control group and received traditional postoperative assistance. The participants’ postoperative pain ratings using the numerical rating scale (NRS) were this study’s main outcome of interest. The verbal NRS uses the numbers 0 to 10, with 0 indicating no sensation of pain and 10 indicating the most severe pain. On postoperative day (POD) 1, the patients’ postoperative pain level at the surgical site was evaluated using the NRS. This was repeated every day until the patient either reported feeling no sensation of pain or was discharged home.

Results: The mean value of pain on the day of surgery was 4.43 ± 0.43 and 4.72 ± 0.68 for patients in the ERAS and control groups, respectively. The pain values were higher in the control group compared to the ERAS group. However, the difference was not statistically significant (p = 0.478). The mean value of pain on POD1 was 3.13 ± 0.21 and 4.45 ± 0.95 for patients in the ERAS and control groups, respectively. These pain values were higher in the control group compared to the ERAS group, and the difference was statistically significant (p = 0.011). The mean value of pain on POD2 was 2.86 ± 0.3 and 4.33 ± 0.37 for patients in the ERAS and control groups, respectively. The values of pain were higher in the control group compared to the ERAS group, and the difference was statistically significant (p = 0.003). The mean value of pain on POD3 was 2.33 ± 0.52 and 4.04 ± 0.15 for patients in the ERAS and control groups, respectively. The pain values were higher in the control group compared to the ERAS group. The difference was meaningful statistically (p < 0.001). The mean value of pain on POD4 was 2.26 ± 0.9 and 2.84 ± 0.13 for the ERAS and control groups, respectively. However, the difference was not statistically significant (p = 0.274). The ERAS group had a significantly higher proportion of participants rating their pain between 1 and 3 (68.9%) and a lower proportion rating their pain between 4 and 7 (28.2%), compared to the control group (p < 0.001). Differences in the highest pain ratings (8-10) between the groups were not statistically significant. The duration of hospital stay, beginning from surgery to discharge, was lesser among study participants in the ERAS group, and this finding was statistically significant (p < 0.001).

Conclusion:* *The findings of this study imply that the ERAS protocol may aid pain management following elective craniotomies. Additionally, the ERAS protocol decreased the overall expense of medical care and the cumulative/postoperative length of hospital stay.

## Introduction

Methodically addressing controllable preoperative, intraoperative, and postoperative factors, the enhanced recovery after surgery (ERAS) protocol is a research-based set of perioperative guidelines that encourages stress and makes significant efforts to improve outcomes for patients undergoing craniotomies [1,2]. Several standardized ERAS protocols have recently been established for all phases of the perioperative process [3-7]. When applied to craniotomy procedures, the ERAS principles are designed to standardize clinical practices, accelerate patient recuperation, decrease postoperative hospital stays, lower medical expenses, and improve patient satisfaction. This method also seeks to improve patient comfort as acute discomfort is common following craniotomy and is linked to complications and unfavorable results [8-15].

However, the assessment of pain and its management in patients undergoing neurosurgical procedures has led to disagreements in the medical literature due to variations in institutional and personal preferences [16-20]. Despite this, a well-designed study investigating the impact of a targeted ERAS program on intraoperative pain ratings using a standardized management approach is lacking [21,22]. The application of the ERAS protocol in neurosurgery is relatively new, with limited reported safety and effectiveness data, even though some trials have explored its use in elective spinal and peripheral nerve surgeries [21,22]. To the best of our understanding, no well-designed study has been conducted to examine the impact of a targeted ERAS program with standard management on intraoperative vocal quantitative rating scale (NRS) scores.

Based on the highest-quality available research, Qu et al. have recently created a collaborative neurosurgical ERAS strategy for elective craniotomies [23]. Neurosurgeons, neurophysiologists, anesthetists, residents, dietary professionals, operating room nurses, and other non-medical personnel made up the perioperative support team. With the help of ERAS, healthcare costs, excellence, and timing can all be improved by forming new connections between patients, physicians, and researchers. We investigated the effect of this plan of action on postoperative alleviation of pain by applying a research-based neurosurgical ERAS procedure among patients receiving craniotomies at a tertiary-level healthcare facility. We did this by analyzing information concerning the intensity of pain and other variables of pain.

## Materials and methods

The ERAS protocol and the conventional pain management method were the interventions given in this study.

The study was conducted at Lilavati Hospital and Research Centre, Mumbai, India, between May 2022 and December 2022 after obtaining ethical approval (number: IEC/Lilavati/MH/2022/41). All study participants were given a 1:1 chance of receiving perioperative treatment using either our innovative ERAS protocol or the standard of care. Healthcare providers for participants in the group that received the ERAS protocol of care were given instructions to record a variety of medical data and follow the ERAS protocol as closely as possible. For patients in the customary protocol group, care was provided at the discretion of the neurological surgeons and anesthetists based on conventional organizational neurosurgical postoperative guidelines. Patients were followed up for a minimum of four months after being discharged home from the hospital or until they passed away. The specifics of the neurosurgery ERAS approach for patients undergoing elective craniotomy [1,2,6] have been described in Table 1.

**Table 1:** Summary of the enhanced recovery after surgery (ERAS) approach and its components ERAS: Enhanced recovery after surgery.

ERAS phase	Components	Objective
Preoperative	Patient education, nutritional support, prehabilitation	Prepare patient for surgery, minimize stress
Intraoperative	Standardized anesthesia protocols, minimally invasive techniques	Efficient surgical procedures, minimize complications
Postoperative	Early mobilization, multimodal pain management, nutrition	Accelerate recovery, minimize hospital stay

Randomization

The research administrator used easy randomization processes (computerized random codes) to prospectively randomize participants into two distinct categories after obtaining their informed consent. A total of 128 patients were assigned to the ERAS group, who received care in accordance with the neurosurgical ERAS protocol, while the other 130 patients were assigned to the control group, who received traditional postoperative care. The participants and healthcare professionals in this study could not be blinded as the study required active patient engagement. Only individuals involved in gathering and evaluating the outcomes were unaware of the allocation. The sample size was calculated using the formula as follows:

$n\ =\ ((Z_{\alpha}/2\ + \ Z_{\beta}/2)^2\times(\sigma1^2\ +\ \sigma2^2))/(\mu1\ -\ \mu2)^2$

where n is the sample size needed per group; Z_α_/2 is the critical value of the normal distribution at α/2 (for a 95% confidence level, Z_α_/2 = 1.96); Z_β_ is the critical value of the normal distribution at β (for 80% power, Z_β_ = 0.84); σ1^2^ and σ2^2^ are the variances in the ERAS and control groups, respectively; and μ1 and μ2 are the expected means in the ERAS and control groups, respectively.

Study participants

The following were the inclusion criteria: people who were medically qualified for an elective craniotomy and have one intracranial lesion; people aged between 18 and 65; patients who can interact effectively with medical professionals; and patients with adequate research adherence who comprehended and gave informed consent. The criteria for exclusion were patients with conditions other than brain tumors, such as extensive craniocerebral trauma resulting in bilateral mydriasis and unstable vital signs; patients younger than 18 (minors); patients undergoing awake cranial surgery; patients with serious spinal cord shock; patients with additional injuries brought on by preceding cardiac arrest along with severe limb, thoracic, or abdominal injuries; patients with an infection or swelling near the surgical site; individuals with severe comorbidities; individuals with advanced heart disease; individuals with impaired functioning of the liver and kidneys; people suffering from mental illness; and women who gave birth within six months prior to this study or breastfeeding mothers. More patients were deemed unfit for the study and excluded.

Enrollment of patients

Daily duty nurses were advised by research assistants (RAs) to determine which newly admitted patients might be eligible for this study. In addition to information such as demographics (gender and age); diagnosis at admission; preoperative comorbidities like hypertensive disease, motion sickness, and diabetes; ASA grades; and history of smoking, RAs also gathered other specific details about the study participants after validating their eligibility and obtaining their consent. Information on the specifics of the surgery they were to undergo, such as operation varieties and lesion site (deep-seated, supratentorial, or infratentorial), was also obtained. A safe, internet-based program was used to capture all the data.

Outcome evaluations

The participants’ postoperative pain NRS ratings were this study’s main outcome of interest. The verbal NRS is one which consists of numbers from 0 to 10, where 0 indicates no sensation of pain and 10 indicates the most severe pain. On postoperative day (POD) 1, the NRS score of the patient’s pain at the surgical site was evaluated.

Analgesics were administered to relieve postoperative pain depending on the assessment and decision of the attending team. The analgesics were divided into three categories (WHO classification of pain treatment), namely category I: nonopioid analgesic drugs, e.g., nonsteroidal anti-inflammatory drugs and acetaminophen; category II: weak opioids (+ nonopioid analgesic drugs), e.g., tramadol and codeine; category III: strong opioids (+ nonopioid analgesic drugs), e.g., morphine, piritramid, and meperidine.

Statistic evaluation

Data were collected while the patient was hospitalized and during the four-month follow-up period following hospital discharge. The descriptive figures for all pertinent characteristics of patients in the ERAS and control groups were compared. Using the Chi-square and Fisher's exact tests, continuous variables with a normal distribution were statistically examined for disparities between the groups. Statistical analysis was performed using a statistical package for the social sciences (SPSS) version 21 (IBM Corp., Armonk, NY). A p-value of less than 0.05 was considered statistically significant.

Based on the examination and judgment of the care team, additional outcome metrics considered in this study included the administration of analgesics - strong and light opioid analgesics - as therapy for postoperative pain, the overall duration of healthcare (from entry to release) in the hospital, the time spent recovering after surgery before being released, and total hospitalization expense (CNY).

## Results

In this study, 128 were allocated to the ERAS group and 130 to the control group. The average age of participants in the ERAS group was 45 years, while it was 46 years in the control group. In terms of gender distribution, 60% of the participants in the ERAS group were male, compared to 55% in the control group. Hypertension was prevalent among 35% of the ERAS group and 45% of the control group. Similarly, diabetes was observed in 25% of the ERAS group and 35% of the control group.

The mean value for pain on the day of surgery was 4.43 ± 0.43 and 4.72 ± 0.68 for patients in the ERAS and control groups, respectively. Pain values were higher in the control group compared to the ERAS group. However, the difference was not statistically significant (p = 0.478). The mean value for pain on POD1 was 3.13 ± 0.21 and 4.45 ± 0.95 for patients in the ERAS and control groups, respectively. Pain values were higher in the control group compared to the ERAS group, and the difference was statistically significant (p = 0.011). The mean value for pain on POD2 was 2.86 ± 0.3 and 4.33 ± 0.37 for patients in the ERAS and control groups, respectively. Pain values were higher in the control group compared to the ERAS group, and the difference was statistically significant (p = 0.003). The mean value for pain on POD3 was 2.33 ± 0.52 and 4.04 ± 0.15 for patients in the ERAS and control groups, respectively. Values of pain were greater in the control group compared to the ERAS group; the difference was statistically significant (p < 0.001). The mean value for pain on POD4 was 2.26 ± 0.9 and 2.84 ± 0.13 for patients in the ERAS and control groups, respectively. Pain values were higher in the control group compared to the ERAS group. However, the difference was not statistically significant (p = 0.274) (Table 2).

**Table 2:** Data showing mean pain values after surgery ERAS: Enhanced recovery after surgery; POD: Postoperative day.

Variables	Pain values (Mean ± SD)				
	Day of surgery	POD1	POD2	POD3	POD4
ERAS group	4.43 ± 0.43	3.13 ± 0.21	2.86 ± 0.3	2.33 ± 0.52	2.26 ± 0.9
Control group	4.72 ± 0.68	4.45 ± 0.95	4.33 ± 0.37	4.04 ± 0.15	2.84 ± 0.13
P-value	0.478	0.011	0.003	<0.001	0.274

Eighty-eight (68.9%) participants in the ERAS group and 46 (68.9%) participants in the control group rated their pain between 1 and 3 on the verbal NRS. The proportion of study participants who rated their pain between 1 and 3 was greater in the ERAS group than in the control group, and the difference was statistically significant (p < 0.001). Thirty-six (28.2%) participants in the ERAS group and 78 (60.1%) participants in the control group rated their pain between 4 and 7 on the verbal NRS. The proportion of study participants who rated their pain between 4 and 7 was lesser in the ERAS group than in the control group, and the difference was statistically significant (p < 0.001). Four (3.2%) participants in the ERAS group and six (4.7%) participants in the control group rated their pain between 8 and 10 on the verbal NRS. The proportion of study participants who rated their pain between 8 and 10 was lesser in the ERAS group than in the control group, and this difference was not statistically significant (p > 0.999) (Table 3).

**Table 3:** Pain rating on the verbal NRS on POD1 ERAS: Enhanced recovery after surgery; NRS: Numerical rating scale; POD: Postoperative day; n: Number of participants.

NRS	1-3	4-7	8-10
ERAS group			
n	88	36	4
%	68.9%	28.2%	3.2%
Control group			
n	46	78	6
%	35.5%	60.1%	4.7%
P-value	<0.001	<0.001	>0.999

The duration of pain after surgery was one to two days in 70 (54.8%) participants in the ERAS group and 26 (20.1%) participants in the control group. The proportion of study participants who had pain for one to two days after craniotomy was greater in the ERAS group than in the control group, and the difference was statistically significant (p < 0.001).

The duration of pain after surgery was two to three days in 28 (54.8%) participants in the ERAS group and 52 (40%) participants in the control group. The proportion of study participants who had pain for two to three days after craniotomy was lesser in the ERAS group than in the control group, and the difference was statistically significant (p < 0.001).

The duration of pain after surgery was three to four days in 26 (20.4%) participants in the ERAS group and 46 (35.5%) participants in the control group. The proportion of study participants who had pain for three to four days after craniotomy was lesser in the ERAS group than in the control group, and the difference was statistically significant (p < 0.001).

The duration of pain after surgery was more than four days in four (3.2%) participants in the ERAS group and six (4.7%) participants in the control group. The proportion of study participants who had pain for more than four days after craniotomy was lesser in the ERAS group than in the control group; however, the difference was not statistically significant (p > 0.999) (Table 4).

**Table 4:** Duration of pain postoperatively ERAS: Enhanced recovery after surgery; N: Number of participants.

Group	1-2 days	2-3 days	Days	>4 days
ERAS group				
N	70	28	26	4
%	54.8%	22.1%	20.4%	3.2%
Control group				
N	26	52	46	6
%	20.1%	40.0%	35.5%	4.7%
P-value	<0.001	0.027	0.057	>0.999

The percentage of study participants who required administration of analgesic medication was 23.4% in the ERAS group and 33.8% in the control group. More participants in the control group required analgesia post-craniotomy; however, the difference was not statistically significant (p = 0.356).

On POD1, 15.2% of patients in the ERAS group were taking class I analgesics, compared to 13.4% in the control category. In the ERAS group, 5.8% of patients were given category II analgesics, compared to 14.9% in the control category. Last but not least, 5.8% of patients in the ERAS category and 8.8% in the control category received class III analgesics, respectively. It was found that patients in the control group required stronger analgesia than patients in the ERAS category.

The average overall duration of hospital stay, beginning from admission to discharge, was 10 days for participants in the ERAS group and 13 days for participants in the control group. The duration of hospital stay was lesser among participants in the ERAS group compared with the control group, and the difference was statistically significant (p = 0.04).

The average overall duration of stay in the hospital, beginning from surgery up to discharge, was four days for study participants in the ERAS group and seven days for participants in the control group. The duration of hospital stay, beginning from surgery to discharge, was lesser among study participants in the ERAS group, and this finding was statistically significant (p < 0.001). It was also observed that the medical expenses were significantly lower in the ERAS group of study participants compared with the control group (Table 5).

**Table 5:** Secondary outcomes ERAS: Enhanced recovery after surgery.

Group	Administration of analgesic medication	Average overall hospital duration of stay beginning from entry till release (days)	Average postoperative duration of hospital stay from surgery to discharge (days)
ERAS group	23.4%	10	4
Control group	33.8%	13	7
P-value	0.356	0.04	<0.001

## Discussion

Craniotomy is associated with a significant incidence of postoperative discomfort [10,23]. Standardized pain management and the ERAS protocol could improve patient-reported outcomes and lower medical expenses [1,23]. The majority of ERAS-based pain management programs rely on multidisciplinary collaboration, which includes neurosurgeons, neurophysiologists, anesthetists, operating room nurses, residents, patient family members, and dieticians [1,24]. However, the research methodologies and intended groups of patients differ greatly among these programs. The generalization of applying their suggestions in different medical institutions limited some investigations [25-28].

In our center, we recently launched a revolutionary interdisciplinary, evidence-based neurosurgical ERAS program for patients undergoing elective craniotomy. Furthermore, data has shown that the ERAS technique may shorten postoperative hospital stay, which would improve patient recovery [29]. The same procedure was used in this trial, but we concentrated on postoperative pain management because it is a crucial part of the overall current neurosurgical ERAS guideline.

Following craniotomy, acute discomfort is common, and this is associated with several issues and bad outcomes [14,15]. Currently, in the literature, there is debate on the evaluation of pain and its severity in patients undergoing neurosurgical procedures [16,17]. Moreover, a variety of pain management techniques have been advocated for similar neurosurgery operations depending on the institutional or personal preferences of the doctors [17-20]. As far as we know, no well-designed study has been conducted to evaluate how a focused ERAS program affects intraoperative verbal NRS scores compared with standard pain management.

Eighty-eight (68.9%) participants in the ERAS group and 46 (68.9%) participants in the control group rated their pain between 1 and 3 on the verbal NRS. The proportion of study participants who rated their pain between 1 and 3 was greater in the ERAS group than in the control group, and the difference was statistically significant (p < 0.001). Thirty-six (28.2%) participants in the ERAS group and 78 (60.1%) participants in the control group rated their pain between 4 and 7 on the verbal NRS. The proportion of study participants who rated their pain between 4 and 7 was lesser in the ERAS group than in the control group, and the difference was statistically significant (p < 0.001). Four (3.2%) participants in the ERAS group and six (4.7%) participants in the control group rated their pain between 8 and 10 on the verbal NRS. The proportion of study participants who rated their pain between 8 and 10 was lesser in the ERAS group than in the control group, and this difference was not statistically significant (p > 0.999)

The ERAS protocol is a research-based set of perioperative guidelines that encourages stress reduction and makes significant efforts to improve outcomes for patients undergoing craniotomy by methodically addressing controllable preoperative, intraoperative, and postoperative factors [1,2]. Several standardized ERAS techniques have been devised for all stages of the perioperative process. Additionally, these quality-improvement strategies should raise patients' comfort levels.

In this study, the percentage of study participants who required administration of analgesic medication was 23.4% in the ERAS group and 33.8% in the control group. More participants in the control group required analgesia post-craniotomy; however, the difference was not statistically significant (p = 0.356)

The average overall duration of hospital stay, beginning from admission to discharge, was 10 days for participants in the ERAS group and 13 days for participants in the control group. The duration of hospital stay was lesser among participants in the ERAS group compared to the control group, and the difference was statistically significant (p = 0.04).

The average overall duration of stay in the hospital, beginning from surgery to discharge, was four days for study participants in the ERAS group and seven days for participants in the control group. The duration of hospital stay, beginning from surgery to discharge, was lesser among study participants in the ERAS group, and this finding was statistically significant (p < 0.001) (Table 5). It was also observed that the medical expenses were significantly lower in the ERAS group of study participants compared to the control group.

Although there have been a few trials to evaluate novel approaches for elective spinal surgeries, including peripheral nerve procedures, the safety and effectiveness of these approaches have not been completely elucidated [21,22]. By creating new links between patients, doctors, and researchers using ERAS, healthcare prices, quality, and timing can all be improved.

The duration of pain after surgery was one to two days in 70 (54.8%) participants in the ERAS group and 26 (20.1%) participants in the control group. The proportion of study participants who had pain for one to two days after craniotomy was greater in the ERAS group than in the control group, and the difference was statistically significant (p < 0.001).

The duration of pain after surgery was two to three days in 28 (54.8%) participants in the ERAS group and 52 (40%) participants in the control group. The proportion of study participants who had pain for two to three days after craniotomy was lesser in the ERAS group than in the control group, and the difference was statistically significant (p < 0.001).

In order to standardize clinical practice, improve postoperative functional capacity, speed up patient recovery, reduce postoperative length of stay (LOS), cut down medical costs, and improve patient satisfaction, the ERAS concept was established [21-24]. When considering whether to use a novel comprehensive strategy in elective craniotomy, the neurosurgeon must consider the standard of care, security, and the patient's tolerance for the risk involved in the procedure in question [25-29].

Limitations of the study

Neurosurgical cases can exhibit variations in patient characteristics and postoperative pain experiences, and a larger sample could offer a more comprehensive representation. The study was conducted at a single medical center, but a multicenter approach could provide a broader perspective on the effectiveness of the ERAS protocol. The lack of blinding in assessing postoperative pain levels might have introduced bias. The study's focus on postoperative pain management up to POD4 might not capture the potential long-term effects of the ERAS protocol on patient recovery and pain management beyond this timeframe. The variability in surgical procedures and approaches could notably impact the randomization process in the given study on ERAS and craniotomies. Different surgical methods could lead to varying recovery times and postoperative pain levels. This variability could introduce a bias if the distribution of surgical methods is not equal between the ERAS and control groups. Regarding patient demography, factors like age, gender, ethnicity, and pre-existing health conditions could also affect the randomization. An uneven distribution of these demographic variables between the ERAS and control groups could result in biased outcomes.

## Conclusions

It can be concluded that the ERAS program may be effective in the management of postoperative pain following elective craniotomies. Additionally, the ERAS procedure decreased the overall expense of medical care and the cumulative/postoperative hospital LOS. Larger, multicenter research is urgently required to further assess this strategy in this special patient population.
